# Effect of Early Rehabilitation during Intensive Care Unit Stay on Functional Status: Systematic Review and Meta-Analysis

**DOI:** 10.1371/journal.pone.0130722

**Published:** 2015-07-01

**Authors:** Ana Cristina Castro-Avila, Pamela Serón, Eddy Fan, Mónica Gaete, Sharon Mickan

**Affiliations:** 1 Carrera de Kinesiología, Facultad de Medicina, Clínica Alemana Universidad del Desarrollo, Santiago, Chile; 2 Internal Medicine Department, Faculty of Medicine, Universidad de La Frontera, Temuco, Chile; 3 Interdepartmental Division of Critical Care Medicine, University of Toronto, Toronto, Canada; 4 Department of Primary Care Health Sciences, University of Oxford, Oxford, United Kingdom; The University of Queensland, AUSTRALIA

## Abstract

**Background and Aim:**

Critically ill survivors may have functional impairments even five years after hospital discharge. To date there are four systematic reviews suggesting a beneficial impact for mobilisation in mechanically ventilated and intensive care unit (ICU) patients, however there is limited information about the influence of timing, frequency and duration of sessions. Earlier mobilisation during ICU stay may lead to greater benefits. This study aims to determine the effect of early rehabilitation for functional status in ICU/high-dependency unit (HDU) patients.

**Design:**

Systematic review and meta-analysis. MEDLINE, EMBASE, CINALH, PEDro, Cochrane Library, AMED, ISI web of science, Scielo, LILACS and several clinical trial registries were searched for randomised and non-randomised clinical trials of rehabilitation compared to usual care in adult patients admitted to an ICU/HDU. Results were screened by two independent reviewers. Primary outcome was functional status. Secondary outcomes were walking ability, muscle strength, quality of life, and healthcare utilisation. Data extraction and methodological quality assessment using the PEDro scale was performed by primary reviewer and checked by two other reviewers. The authors of relevant studies were contacted to obtain missing data.

**Results:**

5733 records were screened. Seven articles were included in the narrative synthesis and six in the meta-analysis. Early rehabilitation had no significant effect on functional status, muscle strength, quality of life, or healthcare utilisation. However, early rehabilitation led to significantly more patients walking without assistance at hospital discharge (risk ratio 1.42; 95% CI 1.17-1.72). There was a non-significant effect favouring intervention for walking distance and incidence of ICU-acquired weakness.

**Conclusions:**

Early rehabilitation during ICU stay was not associated with improvements in functional status, muscle strength, quality of life or healthcare utilisation outcomes, although it seems to improve walking ability compared to usual care. Results from ongoing studies may provide more data on the potential benefits of early rehabilitation in critically ill patients.

## Introduction

Intensive care units (ICUs) and multi-disciplinary team management have evolved improving the survival of critically ill patients [1–3], but these patients still suffer from functional impairments after ICU discharge [4]. Due to the nature of critical illness, medications (e.g., sedation) and devices (e.g., continuous renal replacement therapy) used in the ICU, patients spend a great amount of time immobilized in bed [1,5], leading to physical deconditioning and loss of functionality. Following hospital discharge, ICU survivors have persistent functional impairment and decreased quality of life, attributed to proximal weakness, loss of muscular mass, and fatigue [6]. Even five years after hospital discharge, ICU survivors still report decreased capacity to perform vigorous exercise, as compared to their premorbid condition prior to critical illness [7].

ICU-acquired weakness (ICUAW) is one detrimental effect of critical illness on physical function. This term refers to a wide variety of disorders characterised by acute onset of neuromuscular impairment for which there is no other plausible cause than the critical illness, typically associated with multiorgan failure [8]. One criterion frequently used to diagnose the presence of ICUAW is an assessment of six bilateral muscle groups using the 5-point grade scale developed by the Medical Research Council (MRC). A MRC sum score below 48 (maximum score 60) is diagnostic of ICUAW [8,9]. In the literature, the pooled prevalence of ICUAW has been found to range from 39% (95% confidence interval [CI]: 37–43%. n = 1282) [10] to 46% (95% CI: 43–49%. n = 1421)[11] of patients admitted to an ICU. Female sex, severity of illness, hyperglycaemia, parental nutrition, duration of ICU stay, glucocorticoid use and neuromuscular blocking agents [11] have been associated with ICUAW; while, early physical therapy, intensive insulin therapy, and electrical muscle stimulation [12] have been associated with decreased ICUAW.

In the ICU context, the term ‘mobilisation’ is used to refer to physical activity of sufficient intensity to produce physiological benefits, namely enhanced circulation, central and peripheral perfusion, ventilation, muscle metabolism and alertness. Mobilisation can also prevent deep vein thrombosis and venous stasis [1,3]. Common therapeutic strategies for mobilisation are: passive and active range of motion, active side to side turning, cycling in bed, exercises in bed, sitting on the edge of the bed, transferring from bed to a chair, ambulation, hoist therapy, tilt table, active resistance exercises and electrical muscle stimulation [1,13]. In this review, we have used mobilisation and early rehabilitation as synonyms, to refer to interventions aiming to recover functional status.

Some data suggest that exercise/physical therapy started early may improve physical function in ICU survivors [14] and prevent ICUAW [12]. Currently, the National Institute for Health and Care Excellence [15] and the European Respiratory Society in a joint effort with the European Society of Intensive Care Medicine [1] recommend mobilisation of patients during their stay in critical care units, despite insufficient evidence to support the recommendation. Currently, there is one systematic review on interventions to improve physical function in ICU survivors [14], one on interventions to prevent ICUAW [12] and three on the effect of early rehabilitation in mechanically ventilated and ICU patients [5,16,17]. They report a positive effect on physical function for rehabilitation started in the ICU, but these studies have not used an established minimum definition of usual care and they have not included the time at which intervention was started or the dose of physical activity. Thus, this systematic review aims to determine the impact of time from ICU admission to first mobilisation session and the dose of physical therapy on functional status. A secondary objective is to describe the current interventions available in this clinical setting which could be implemented in addition to usual care.

## Methodology

A systematic review of the literature was performed to determine the effect of early rehabilitation/ mobilisation on the functional status in patients admitted to the ICU or HDU. The protocol for this systematic review was registered at PROSPERO (CRD42013004535). This manuscript adheres to the PRISMA statement recommendations (see S1 PRISMA Checklist).

### Selection Criteria

We performed an electronic search of Medline, Pubmed, EMBASE, CINAHL, AMED, PEDro, Cochrane Library, Cochrane Central Register of Controlled Trials, ISI Web of Science (including Science Citation Index Expanded and Conference Proceedings Citation Index), Clinicaltrials.gov, WHO International Clinical Trials Registry Platform (ICTRP), IFPMA Clinical Trials Portal, Current Controlled Trials, Scielo and LILACS from inception to April 1^st^, 2014. Grey literature was identified using OpenSIGLE.com. The search strategy included free text words (critical care, intensive care units, critical illness, intensive care, ICU, HDU, rehabilitation, physical therapy modalities, exercise therapy, physical exertion, early ambulation, muscle weakness/rehabilitation, muscle weakness/therapy, neuromuscular diseases/ rehabilitation and recovery of function) and controlled vocabulary adapted for every database (see S1 Supporting Information). Also, the randomised controlled trial (RCT) search filter from the Cochrane Handbook [18], SIGN methodological filter [19] and Manriquez (2008) filter for LILACS [20] were employed. No language or date limits were used. The reference lists of the systematic reviews published in the topic were hand searched in order to identify articles that could be included in this review. Authors of eligible studies were contacted to ask for clarification of methodological details and results in the case of unpublished/missing data.

Eligible studies were randomised or controlled clinical trials comparing rehabilitation to usual care in ICU/HDU patients. Adult patients had to be admitted to ICU/HDU for at least 48 hours and be followed for outcomes until ICU discharge.

We excluded studies that: compared passive therapies (i.e., not involving conscious muscle activation) to usual care; started rehabilitation after ICU/HDU discharge; evaluated interventions in the same patient (e.g., electrical stimulation is applied in one limb and the other serves as control); enrolled more than 20% of patients under 18 years; or had patients admitted to an ICU/HDU due to neurological conditions (e.g., stroke, acquired or traumatic brain injury, multiple sclerosis, amyotrophic lateral sclerosis, brain tumour, spinal cord injury, neuromuscular diseases), or trauma that could limit rehabilitation (e.g., major trauma, fractures, joint replacement).

#### Types of Interventions

***Usual care***: encompassed passive range of motion, position change every two hours while the patient was unconscious and once they regained consciousness, it aimed to increase the level of activity, first in an active-assisted way with exercise in bed, progressing to sitting on the edge of bed, then transition from sitting to standing to finally reach ambulation (assisted or independent) [21–23]. Usual care had to incorporate an active element at some point of ICU stay, in order to be included.

***Rehabilitation/mobilisation***: could have been led by a physiotherapist, occupational therapist or other health professional. It was expected that intensity, frequency and duration of the rehabilitation was tailored to the patients need and physiological stability, although a standardised protocol could have been used. Rehabilitation/mobilisation programs should have included three or more of the following therapeutic strategies: passive and active range of motion, active side to side turning, cycling in bed, exercises in bed, sitting on the edge of the bed, transferring from bed to a chair, marching on the spot, ambulation, hoist therapy, tilt table, active resistance exercises and electrical muscle stimulation [1,13]. In order to be considered rehabilitation, the intervention should have included more components than usual care, be performed at a higher dose (intensity, volume or frequency) or started at an earlier point than usual care. The main objective of rehabilitation should have been functional recovery.

#### Types of outcome measures

The primary outcome was a measure of functional status at ICU discharge, using instruments that assess function of lower and upper body during functional tasks (e.g., functional independence measure [FIM], Barthel index, Katz activities of daily living or physical function in the ICU test [PFIT]) [24]. Secondary outcomes were: walking ability (e.g., 6 minute walk test [6MWT], timed-up-and-go test or ability to walk independently), muscle strength (e.g., MRC sum score, handgrip strength, handheld dynamometry or ICUAW), quality of life (e.g., Medical Outcomes Study Short Form-36 [SF-36] or European quality of life-5 domains [EQ-5D]), duration of mechanical ventilation, length of stay (ICU and hospital) and time in rehabilitation after hospital discharge. When available, these data were collected at initial evaluation, at ICU and hospital discharge, and at three, six and twelve months after hospital discharge.

### Methods of the review

#### Selection of studies

The initial screening of titles was performed by two independent reviewers (ACCA and MG). Disagreements were resolved by consensus. The citations were classified as eligible, uncertain about eligibility, or excluded and stored in an Excel spreadsheet (Microsoft Corporation, Redmond, WA). Abstracts of references considered relevant based on the title were then checked by two independent reviewers (ACCA and SM). The full text articles of eligible and uncertain about eligibility citations were retrieved and reviewed. If, after reviewing the full text version of the article, eligibility was not clear or there was missing information, the authors were contacted by email. When there was no response, a reminder email was sent at two week intervals. After three unsuccessful attempts at contact, the decision was made based on the information available.

#### Data extraction

Data were extracted by the main author (ACCA) and then independently checked by two other reviewers (EF and PS). Disagreements were resolved by consensus. Data were extracted about the study design, aspects of the methodological quality, number of individuals randomised and analysed, patients’ baseline characteristics, primary and secondary outcome results. When the information was extracted from a graph, data points were obtained using GetData Graph Digitizer (www.getdata-graph-digitizer.com).

#### Assessment of methodological quality

The PEDro scale was used to assess the methodological quality of the included studies [25]. Every criterion was marked as present or absent, and a score was calculated with a maximum of 10 points.

### Data analysis

Meta-analysis was performed when data were presented for the same outcome, using the same measurement tool, in at least two different clinical trials, using Review Manager (RevMan) version 5.2 (Copenhagen: The Nordic Cochrane Centre, The Cochrane Collaboration, 2008) software. In the case of categorical variables, the estimates are shown as risk ratio (95% confidence intervals). For continuous variables, when mean (standard deviation [SD]) for change from baseline was not available (or median [interquartile range]) provided, authors were contacted in order to obtain these data. If the data were not obtained, they were imputed using the method described in the Cochrane Handbook [26] which requires that at least one of the studies reports standard deviations for change from baseline. When no information on standard deviations were available, the data were not pooled and only included in the narrative synthesis. Continuous variables were compared using t-tests. A fixed-effect model was used because it provides better estimates when few studies are included [27]. The statistical heterogeneity was quantified using I^2^ test, considering a threshold of 50% for high statistical heterogeneity [28].

#### Subgroup and Sensitivity analyses

A number of predefined subgroup analyses were planned: comparisons between patients with and without ICUAW, early (≤ 7 days) versus delayed (>7 days) start of rehabilitation since ICU admission, and low (≤150 min/week) versus high (>150 min/week) volume of rehabilitation. However, we were unable to perform pooled analyses due to limited data. As a result, the information on these subgroups is presented in the narrative synthesis.

In order to explore if the methodological quality was a cause of observed heterogeneity, a sensitivity analysis was carried out. High methodological quality was defined as fulfilling four quality criteria: randomisation assignment, concealed allocation, blinding of outcome assessor and intention-to-treat analysis. An article was considered of low methodological quality if it did not fulfil one or more of the high quality criteria. PEDro scale scores were calculated, but not used as measurement of methodological quality, because higher scores do not always imply better quality and the four criteria before mentioned have been related to overestimation of intervention effect size [29].

## Results

### Study selection

**Fig 1:**
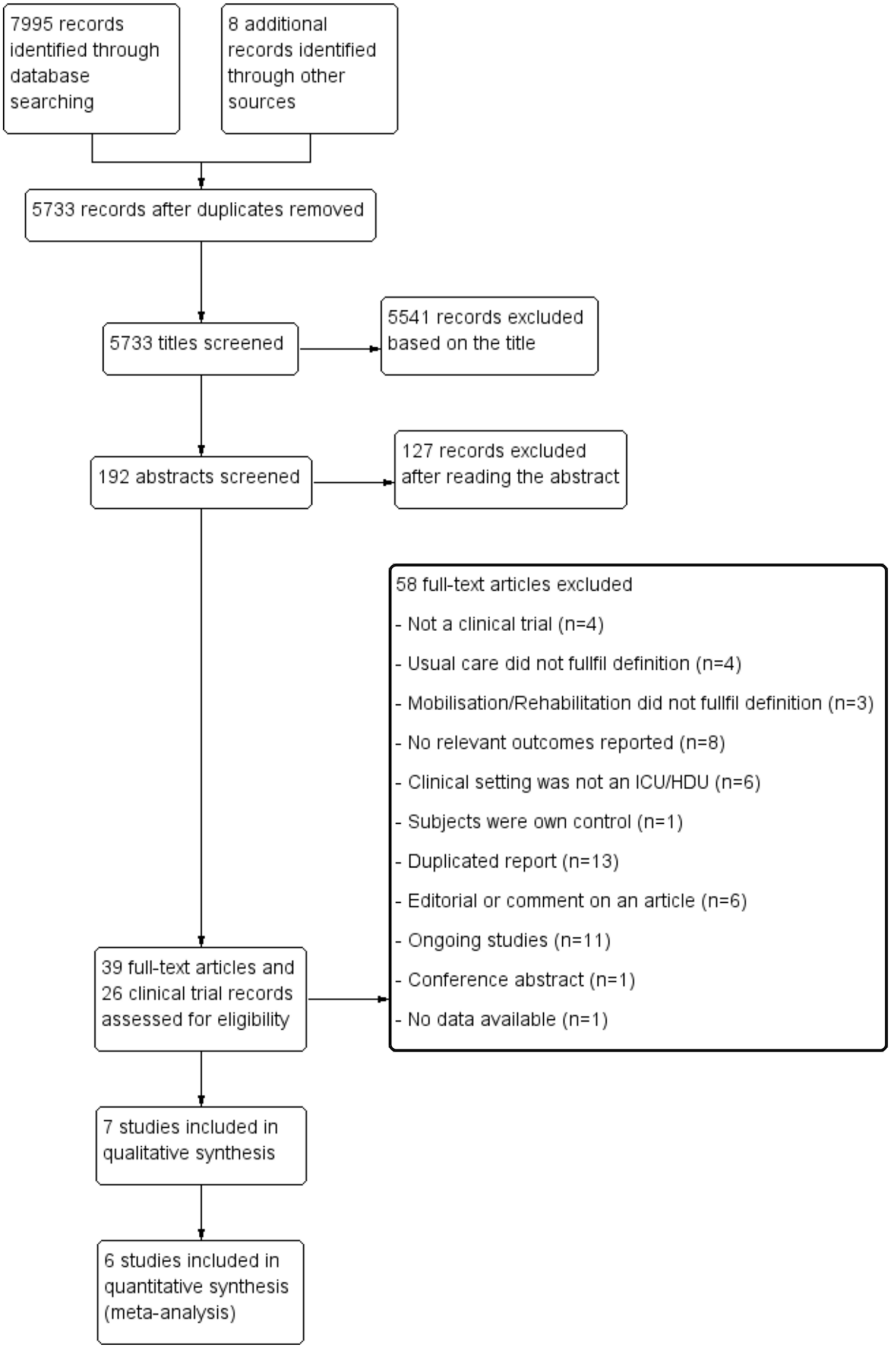
PRISMA flow diagram.

The search in all databases included in this systematic review yielded 5733 articles. One hundred and ninety two abstracts were screened and 65 full texts were read. Thirty-nine articles and 26 clinical trial registry entries were reviewed in full (Fig 1). Characteristics about the population, description of the intervention and control group and outcomes measured in ongoing trials are shown in S1 Table. Details about studies and reasons for exclusion can be found in S2 Table. Seven studies were included in the qualitative synthesis [30–36], but only six were included in the meta-analysis [30–35].

### Methodological quality

Details about methodological quality assessment using the PEDro scale can be found in Table 1. Six trials used a randomised method for allocation [30–33,35,36], and one used a time block sequential design [34]. Only four trials described an adequate concealment of the randomisation schedule [31,32,34,36]. In three trials, subjects were not comparable at baseline [31,33,35]. Blinding of patients was possible in one trial [34]. None of the studies included was able to fulfil the criterion of blinding of the therapist. Blinding of the outcome assessors was achieved in four trials [32,34–36]. Only two trials had an adequate follow-up [32,34]. Intention-to-treat analysis was carried out in five trials [32–36]. All trials reported between-group comparisons and point estimates with variability measures for at least one key outcome. Three trials were considered as having a high methodological quality based on the fulfilment of a random allocation, concealed assignment, blind outcome assessor and intention-to treat analysis [32,35,36].

**Table 1:** Quality assessment based on PEDro scale of clinical trials included in the systematic review.

	Nava 1998	Schweickert et al 2009	Burtin et al 2009	Routsi et al 2010	Hanekom et al 2012	Denehy et al 2013	Brummel et al 2014	Studies meeting criterion, n (%)
Eligibility criteria	Yes	Yes	Yes	Yes	No	Yes	Yes	5 (71.4)
**Randomised allocation**	**Yes**	**Yes**	**Yes**	**Yes**	**No**	**Yes**	**Yes**	**6 (85.7)**
**Concealed allocation**	**No**	**Yes**	**Yes**	**No**	**No**	**Yes**	**Yes**	**4 (57.1)**
Comparable at baseline	Yes	Yes	No	No	Yes	No	Yes	4 (57.1)
Blinded subjects	No	No	No	No	Yes	No	No	1 (14.3)
Blinded therapists	No	No	No	No	No	No	No	0
**Blinded assessors**	**No**	**Yes**	**No**	**No**	**Yes**	**Yes**	**Yes**	**4 (57.1)**
Adequate follow-up	No	Yes	No	No	Yes	No	No	2 (28.6)
**Intention-to-treat analysis**	**No**	**Yes**	**No**	**Yes**	**Yes**	**Yes**	**Yes**	**5 (71.4)**
Between-group comparisons	Yes	Yes	Yes	Yes	Yes	Yes	Yes	7 (100)
Point estimates and variability	Yes	Yes	Yes	Yes	Yes	Yes	Yes	6 (100)
Total Score	4/10	8/10	4/10	4/10	7/10	6/10	7/10	

### Participants

In total, 774 individuals participated in the clinical trials included in this review (Table 2). Of those, 419 (54%) belonged to the intervention group and 355 (46%) to the control group. Two hundred and ninety-three (38%) were females. Patients admitted with ARDS, pneumonia or sepsis constituted the largest diagnostic category (38%), followed by elective or emergency surgical admission (30%) and chronic heart failure, heart disease or cardiogenic shock (12%). The studies were carried out in a medical ICU in three studies [31,32,36], surgical ICU in three studies [31,34,36], multidisciplinary ICU for two studies [33,35] and one respiratory ICU [30]. These studies took place in the United States [32,36], Belgium [31], Italy [30], Greece [33], South Africa [34] and Australia [35].

**Table 2:** Demographic characteristics of patients of included studies in the systematic review.

Reference	Group	Sample size (n)	Age, mean ± SD or Median (IQR)	Sex, n (%) females	APACHE II‡, mean ± SD or Median (IQR)	Admission diagnosis*, n (%)	
Schweickert et al 2009						Acute Lung Injury	58 (55.8)
	Intervention	49	57.7 (36–69)	29 (59)	20.0 (16–24)	COPD/Asthma exacerbation	19 (18.3)
	Usual Care	55	54.4 (47–66)	23 (42)	19 (13–23)	Sepsis	16 (15.4)
						Malignancy	3 (2.9)
						Haemorrhage	3 (2.9)
Burtin et al 2009§						Cardiac surgery	28 (31.1)
	Intervention	31	56 ± 16	9 (29.03)	26 ± 6	Transplant surgery	18 (20)
	Usual Care	36	57 ± 17	10 (27.8)	25 ± 4	Thoracic surgery	11 (12.2)
						Medical diagnosis	19 (21.1)
Nava 1998	Intervention	60	65 ± 6	22 (36.7)	Not Reported	COPD exacerbation	80 (100)
	Usual Care	20	67 ± 9	7 (35)	Not Reported		
Routsi et al 2010						Post-surgical	25 (26.9)
	Intervention	44	63 ± 20	14 (31.8)	18 ± 4	Trauma	26 (28)
	Usual Care	49	59 ± 20	12 (24.5)	19 ± 5	Sepsis/septic shock	25 (26.9)
						Respiratory failure	6 (6.4)
Denehy et al 2013						Cardiac surgery	34 (22.7)
						Pneumonia	26 (17.3)
	Intervention	74	61.4 ± 15.9	31 (41.9)	19 ± 6	Other surgery	23 (15.3)
	Usual Care	76	60.1 ± 15.8	24 (31.6)	20.7 ± 7.7	Cardiac disease	17 (11.3)
						Cardiac arrest	8 (5.3)
						Liver disease/transplant	15 (10)
						Sepsis	13 (8.7)
Hanekom et al 2012	Intervention	96	52.1 (18.5)	37 (39)	18.4 ± 27.4	Elective surgery	110 (57)
	Usual Care	97	50.2 (17.9)	37 (38)	16.2 ± 22.7	Emergency surgery	29 (15)
						Trauma	32 (16.6)
Brummel et al 2014						Sepsis/ARDS/Pneumonia	52 (59.8)
	CT	43	62 (54–69)	15 (35)	25 (19.5–29.5)	Abdominal surgery	13 (14.9)
	Early PT	22	62 (48–67)	9 (42)	21.5 (20–28.8)	Airway protection	8 (9.2)
	Usual Care	22	60 (51–69)	14 (64)	27 (17.5–31)	Cirrhosis/GI bleeding	4 (4.6)
						CHF/Arrhythmia/ Cardiogenic Shock	2 (2.3)

^‡^ APACHE II: Acute physiological and chronic health evaluation II. It measures severity of disease. It is assessed within 24 hours since admission to intensive care. Scores range from 0 to 71 and are associated to a predicted mortality. 25–29 points have predicted mortality of 55% for non-operative admission and 35% post-surgery; ≥35 points have 85% and 88% predicted mortality for non-surgical and post-surgical admission, respectively [51].

*Only most prevalent admission diagnoses for every study are reported in this table.

^§^Admission diagnosis were reported for total number of patients randomised (n = 90).

I: Intervention group; C: Control Group; CT: Cognitive therapy; ARDS: Acute respiratory distress syndrome; GI: Gastrointestinal; CHF: Chronic heart failure.

### Interventions

A detailed description of the design, eligibility criteria, intervention and control groups for each trial included is reported in Table 3. Frequency of early mobilisation ranged from once daily [32–36], to five times a week [31] (Table 4). The frequency of usual care ranged from one or two sessions a week [36] to equal or more than five times a week [30,31,34,35]. Two studies did not report frequency of usual care [32,33]. In relation to the length of each rehabilitation session, two studies reported the actual duration [32,36], four trials presented a fixed duration [30,31,33,35] and one did not provide information on the duration of each session [34]. One article presented the actual duration of sessions [32], one study reported a fixed length for each session [30] and the other five trials did not give data about duration of sessions for the control group [31,33–36]. None of the studies included in this systematic review reported information about intensity for either group.

**Table 3:** Description of design, population, setting and details about intervention and control group for included studies.

				Description	
Reference	Design	Population	Clinical Setting	Intervention	Usual Care
Schweickert et al 2009	Multicentre parallel randomised controlled trial	Mechanical ventilation (MV) for < 72 hrs., expected to continue for at least 24 hrs., functionally independent before admission (Barthel index > 70 obtained from a proxy)	Medical ICU in Chicago and Iowa, Unites States	PT + OT + interruption of sedation. Sessions started with passive range of motion and when patient was able to interact, it progressed to active assisted and active range of motion exercises in supine and sitting in the bed.	Standard medical and nurse care. Physical and occupational therapy as ordered by primary care team
Burtin et al 2009	Single centre parallel	At least five days in ICU, stable cardio	Medical and Surgical ICU in	Usual Care + Cycloergometer. Sedated patients: it was used as passive mobiliser at 20 cycles/min.	Respiratory physiotherapy and standardised
	randomised controlled trial	respiratory condition, expected ≥7 days more in the unit	Leuven, Belgium	Cooperative patients: two sessions of 10 min each of active cycling	mobilisation sessions of upper and lower limbs
Nava 1998				Four levels progressive mobilisation program.	
	Single centre	Patients with COPD		Step I: sitting upright in bed or chair and active or passive range of motion.	Standard medical
	parallel randomised	admitted to RICU after an acute	Respiratory ICU in Montescano,	Step II: progressive walking retraining	and nurse care. Also Steps I and II of
	controlled trial	respiratory failure episode. Clinically stable.	Italy	Step III: Respiratory muscle training (RMT) with threshold device, cycling and climbing 25 steps in a stair 5 times.	mobilisation program
				Step IV: Treadmill.	
Denehy et al 2013	Single centre parallel randomised controlled trial	Five or more days in ICU, intensive care specialist agreed with their participation	ICU in Melbourne, Australia	Exercise sessions based in baseline PFIT, including sitting out of bed, sit to stand, marching on the spot and shoulder elevation. Rehabilitation continued in the general ward, but the intensity was adjusted according to 6MWT results	Usual Care
Hanekom et al 2012	Single centre sequential time-block clinical trial	Patients requiring support/ monitoring after elective/ emergency surgery	Surgical ICU in Stellenbosh, South Africa	Protocol-based intervention, including one algorithm for each of the following conditions: Upper abdominal surgery, rehabilitation for chronic ventilated patients, thoracic injuries, acute lung injury, pulmonary dysfunction.	Decisions related to activities and intervention frequency were based on clinical decision of the therapist responsible for patient care.
Routsi et al 2010	Single centre parallel randomised controlled trial	Two days in the unit with APACHE II score ≥ 13 points	Multi-disciplinary ICU in Athens, Greece	Usual care+ electrical muscle stimulation in vastus lateralis, vastus medialis and peroneous longus of both lower limbs. They used biphasic, symmetric impulses of 45 Hz, 400 μsec pulse duration, 12 seconds on (0.8 second rise time and 0.8 second fall time) and 6 seconds off.	Standard medical and nurse care. Physiotherapy care included passive range of motion, sitting out of bed, transferring from bed to chair and sitting on a chair.
Brummel et al 2014	Single centre parallel	Patients admitted for respiratory failure, cardiogenic	Surgical and Medical ICU in	Early PT: Active mobilisation, sitting out of bed, standing and ambulation	Usual Care as
	randomised controlled trial	shock, haemorrhagic shock, and/or septic shock. Clinically stable	Nashville, United States	Early PT + Cognitive therapy (CT): it also included exercises to improve orientation, attention and memory	ordered by treating clinician

ICU: Intensive care unit; RICU: Respiratory intensive care unit; COPD: Chronic obstructive pulmonary disease; MV: Mechanical ventilation; PT: physiotherapy/physical therapy; OT: Occupational therapy; CT: Cognitive therapy; PFIT: Physical function in ICU test; 6MWT: 6-minute walking test.

**Table 4:** Description of frequency, duration, intensity and cumulative time a week for intervention and usual care group.

Reference	Group	Frequency	Duration	Intensity	Cumulative time in a week	Time since admission to first mobilisation session (days)
Schweickert et al 2009	Intervention	Daily sessions	Duration in MV (min): 19.2 (10.2–28.8)	Individually adjusted	Median of 134.4 min/week in MV	1.5 (1–2.1)
			Duration after weaning (min): 12.6 (4.8–19.8).	intensity	and 88.2 min/week after weaning.	
	Usual Care	Not reported	Duration in MV (min): 0 (0–0)	Not reported	Not calculable	7.4 (6–10.9)
			Duration after weaning (min): 11.4 (0–22.8).			
Burtin et al 2009	Intervention	Five times a week	20 min	Individually adjusted intensity	100 min/week.	14 ± 10
	Usual Care	Five times a week.	Not reported	Not reported	Not calculable	10 ± 8
Nava 1998	Intervention	Two daily sessions.	30 to 45 min each session. In Level III, 10 min of RMT and 20 min of cycling. For level IV, 30 min of treadmill.	In level III, RMT at 50% MIP and cycling with 15 watts of load. In level IV, intensity was at 70% of load	420 to 630 min/week.	Not Reported
	Usual Care	Two daily sessions	30 to 45 min each session.	Not reported	420 to 630 min/week	Not Reported
Denehy et al 2013	Intervention	Daily sessions	15 min during ICU stay progressing to 30 min in general ward and up to 60 min before discharge.	Individually adapted intensity	105 min/week in ICU, 210 min/ week in general ward and 420 min/week before discharge	Not Reported
	Usual Care	Daily sessions	Not reported	Not reported	Not calculable	Not Reported
Hanekom et al 2012	Intervention	Daily sessions	Based on the protocol	Based on the protocol	Not calculable	0.58 ± 0.29
	Usual Care	During weekdays. Patients in most need received sessions during weekends.	Based on therapist decision	Based on therapist decision	Not calculable	1.13 ± 0.83
Brummel et al 2014	Early PT	Once daily.	Nurse or physician led sessions: 15 (10–20) min. PT/OT led sessions: 23 (16–26) min.	Not reported	Median of 105 to 161 min/week	1 (1–1)
	Cognitive Therapy	Twice daily.	20 min+ early PT	Not reported	140 min/week of CT + median of 105 to 161 min/week of PT	1 (1–1.8)
	Usual Care	1–2 sessions per week.	Not reported	Not reported	Not calculable	3 (2–6)
Routsi et al 2010	Intervention	Daily sessions	55 min	Individually adjusted intensity to reach visible contraction	384 min/week	2 ± 0
	Usual Care	Not reported	Not reported	Individually adjusted intensity	Not calculable	Not Reported

MV: Mechanical ventilation; PT: physiotherapy/physical therapy; CT: Cognitive therapy; OT: Occupational therapist; RMT: Respiratory muscle training; MIT: Maximal inspiratory pressure.

Two studies could be regarded as delayed (≥7 days from ICU admission) start [30,31] and two categorised as early (< 7 days) start [34,36]. For Schweickert et al. [32] and Routsi et al. [33] the intervention group would be labelled as early start and the control group as delayed in the case of Schweickert et al [32]. Routsi et al [33] did not report data for the control group. Only one trial did not give information about time from admission until first session [35]. We considered patients recruited into the study by Nava [30] as a delayed start of rehabilitation, since they were transferred from an ICU to the respiratory ICU when they were stable.

It was possible to calculate the volume (mean or median duration*frequency) of rehabilitation and/or usual care for six trials [30–33,35,36]. Only one study performed more than 150 min/week for both groups [30]. In the study by Brummel et al. [36], a higher volume of rehabilitation was achieved when sessions were led by a physical/occupational therapist, but not when they were led by a nurse or physician. The other four studies performed less than 150 min/week during the ICU stay [31–33,35]. Five of the articles included did not present enough information to calculate volume of physical activity for the control group [31–36].

### Effects of Interventions

#### Functional status

Three studies measured functional status [32,35,36]. Only the study by Schweickert et al [32] demonstrated a significant benefit of early rehabilitation on functional status (Barthel index) at hospital discharge (Table 5).

**Table 5:** Summary of results for outcome functional status.

			Mean ± SD or Median (IQR)		
Reference	Instrument	Time point	Intervention	Control	p-value
Schweickert et al 2009	Barthel index¥	Hospital discharge	75 (7.5–95)	55 (0–85)	0.05
Brummel et al 2014	Katz activities of	Hospital	CT: 3 (1–6)	1 (0–2.8)	0.25
	daily living ¥	discharge	PT: 0.5 (0–4.5)		
Denehy et al 2013	Physical Function in ICU test (PFIT) ¥	ICU discharge	7.7 ± 1.7	8 ± 1.5	0.32

CT. Cognitive therapy plus early physiotherapy. PT: Early physiotherapy.

^¥^: Higher scores are related to better performance

#### Walking ability

Five studies reported walking ability [30–32,35,36]. Three trials reported that the intervention group walked a longer distance than patients receiving usual care [30–32]. It is noteworthy that in the trial by Denehy et al [35] there was a significant difference in 6-minute walk test distance in favour of usual care (187.9 ± 126.1 meters) as compared with the intervention group (146.4 ± 79.4 meters) at ICU discharge (p = 0.02).

Although two studies showed no difference in the timed-up-and-go test at hospital discharge and three months follow-up [35,36], Denehy et al [35] found a significant benefit in favour of usual care at hospital discharge (Table 6).

**Table 6:** Summary of results for outcome walking ability.

			Mean ± SD or Median (IQR)		
Reference	Instrument	Time point	Intervention	Control	p-value
Schweickert et al 2009	Distance walked without assistance (meters)	Hospital discharge	33.4 (0–91.4)	0 (0–30.4)	0.004
Burtin at al 2009	6 min walking test (meters)	Hospital discharge	196 (126–329)	143 (37–226)	<0.05
Nava 1998	6 min walking test (meters)	Hospital discharge	216.8 ±119.9	142.4 ± 77.7	0.02
Denehy et al 2013	6 min walking test (meters)	Hospital discharge	244.2 ± 124	266.7 ± 136.8	0.35
Brummel et al 2014	Timed-up-and-go	Hospital	CT: 17 (11–27)	33 (18.5–68.5)	0.2
	(seconds) ¦	discharge	PT: 16 (12–22)		
Denehy et al 2013	Timed-up-and-go (seconds) ¦	Hospital discharge	18.8 ± 12.2	12.9 ± 6.6	<0.01
Brummel et al 2014	Timed-up-and-go	3 months	CT: 11 (9–13)	8 (8–13)	0.79
	(seconds) ¦	follow-up	PT: 10 (8–13)		
Denehy et al 2013	Timed-up-and-go (seconds) ¦	3 months follow-up	12.2 ± 10	11.6 ± 11.2	0.77

CT. Cognitive therapy plus early physiotherapy. PT: Early physiotherapy.

^¦^: Longer time are related to worse performance

**Fig 2:**
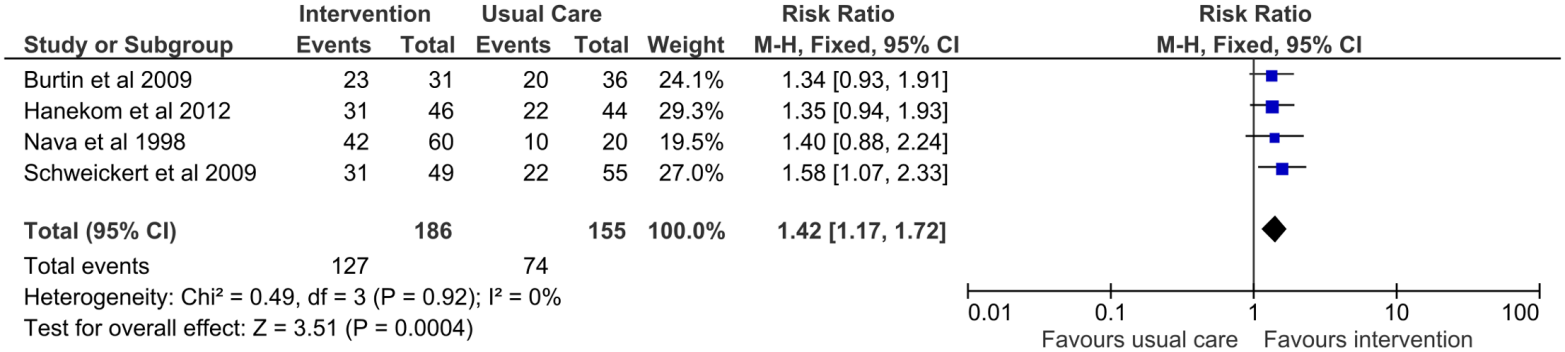
Forest plot for walking without assistance at hospital discharge.

**Fig 3:**
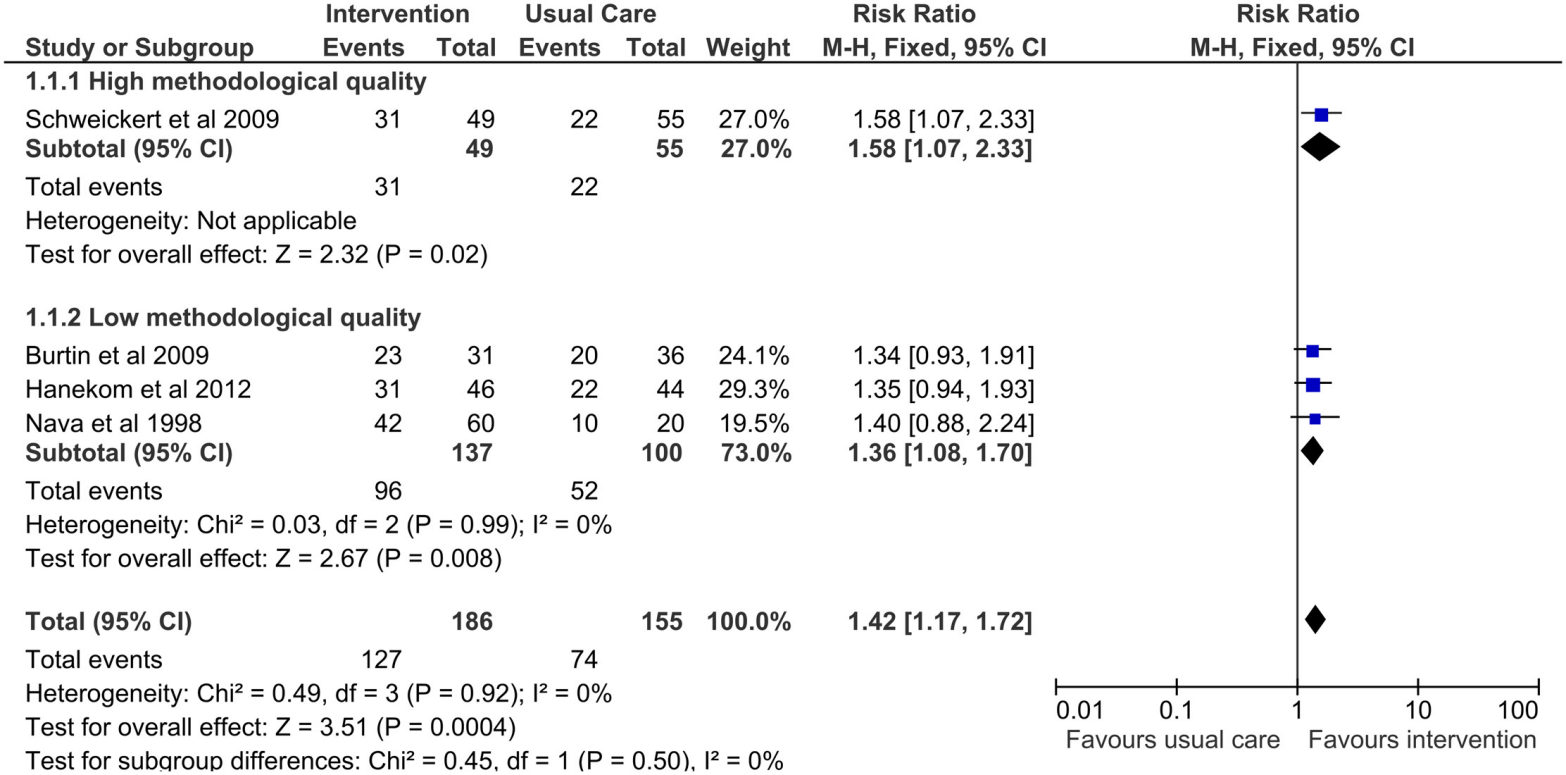
Forest plot for subgroup analysis of walking without assistance at hospital discharge according to methodological quality.

Four studies [30–32,34] measured walking without assistance at hospital discharge and demonstrated a significant benefit with early rehabilitation (pooled risk ratio 1.42; 95% CI: 1.17–1.72) (Fig 2). This result was robust in a sensitivity analysis restricted to studies of high methodological quality (Fig 3).

#### Muscle strength

Two studies reported outcomes measuring peripheral muscle strength [31,32] (Table 7). Although Burtin et al [31] did not find a significant difference in handgrip strength as a percentage of predicted force between intervention and control groups, they did find a significant difference in isometric quadriceps strength measured using handheld dynamometry. Similarly, Schweickert et al [32] reported no significant differences in handgrip strength or MRC sum scores between groups.

**Table 7:** Summary of results for the outcome muscle strength.

			Mean ± SD or Median(IQR)		
Reference	Instrument	Time point	Intervention	Control	p-value
Burtin et al 2009	Handgrip strength (percentage predicted)	ICU discharge	46±20	47±11	0.83
Schweickert et al 2009	Handgrip strength (Kg*force)	Hospital discharge	39 (10–58)	35 (0–57)	0.67
Schweickert et al 2009	MRC score	Hospital discharge	52 (25–58)	48 (0–58)	0.38
Burtin et al 2009	Handheld dynamometry (Isometric Quadriceps strength)	Hospital discharge	2.37±0.62	2.03±0.75	0.05

**Fig 4:**
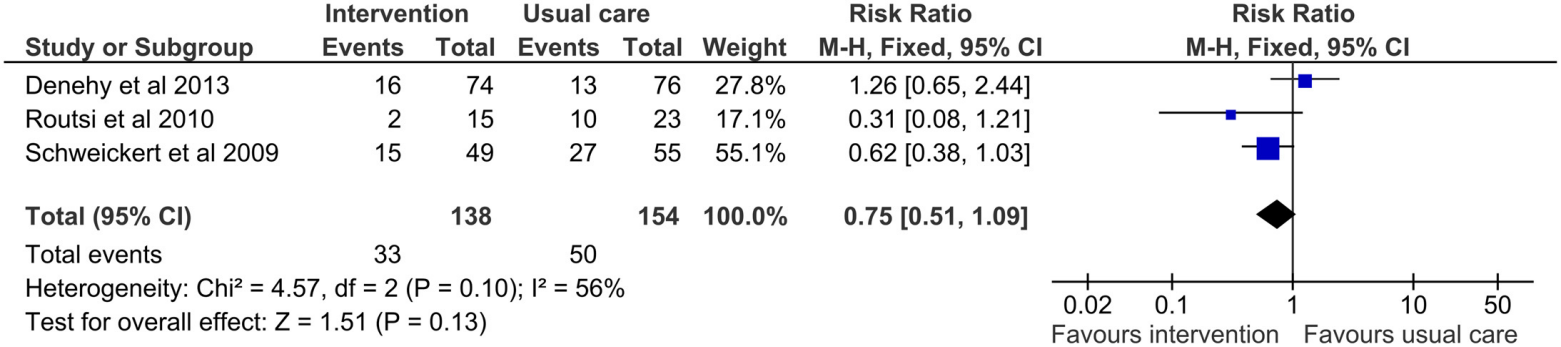
Forest plot for ICU-acquired weakness.

Three trials reported ICUAW as an outcome measure [32,33,35] (Table 8). There was a non-significant association between early rehabilitation and decreased risk of ICUAW (pooled risk ratio 0.75; 95% CI: 0.51–1.09). However, it must be noted that timing of assessment was different in these three trials (Fig 4).

**Table 8:** Summary of results for the outcome ICU acquired weakness.

		Proportion of Events		
Reference	Time point	Intervention	Control	p-value
Denehy et al 2013	Baseline	16/74	13/76	0.48
Routsi et al 2010	When patient was able to cooperate	2/15	10/23	0.05
Schweickert et al 2009	Hospital discharge	15/49	27/55	0.09

#### Quality of life

Two studies reported quality of life as measured by the SF-36 Physical Functioning Subscale [31,35] (Table 9). Only one of them found significant results favouring the intervention group [31], however patients in both groups reported improved scores for quality of life. In contrast, Denehy et al [35] reported that patients in both groups had a lower quality of life than the population mean.

**Table 9:** Summary of results for the outcome quality of life.

			Mean ± SD or Median(IQR)		
Reference	Instrument	Time point	Intervention	Control	p-value
Burtin et al 2009	SF-36 Physical Functioning	Hospital discharge	21 (18–23)	15 (14–23)	<0.01
Denehy et al 2013*	SF-36 v2 Physical Functioning	3 months follow-up	39.9 ± 14.4	42.3 ± 12	0.36

* Values are norm-based t-scores where population mean is 50 and standard deviation is 10.

#### Duration of mechanical ventilation

Two trials reported the effect of early rehabilitation on the duration of mechanical ventilation [32,33]. Schweickert et al [32] found that the intervention group spent significantly fewer days receiving ventilatory support compared to the control group (median [IQR]: 3.4 [2.3–7.3] vs. 6.1 [4.0–9.6], p = 0.02); while, Routsi et al [33] noted a reduction in number of days, but this difference was not statistically significant (median [min-max]: 6 [4–18] vs. 9 [3–25], p = 0.28).

#### Length of stay in ICU and in Hospital

Six trials reported information regarding length of stay in ICU [30–34,36] (Table 10). None found a significant difference between intervention and control groups. Four studies specifically measured length of stay in hospital: despite shorter hospital length of stay in three trials [31,34,36], the differences were not significant between groups.

**Table 10:** Summary of results for length of stay in ICU and length of stay in hospital.

	Length of stay in ICU (days)			Length of stay in hospital(days)		
Reference	Intervention	Control	p-value	Intervention	Control	p-value
Schweickert et al 2009	5.9 (4.5–13.2)	7.9 (6.1–12.9)	0.08	13.5 (8.0–23.1)	12.9 (8.9–19.8)	0.93
Burtin et al 2009	25 (15–37)	24 (17–34)	0.14	36 (28–47)	40 (28–49)	0.15
Nava 1998	38.1 ± 14.3	33.2 ± 11.7	>0.05	Not reported	Not reported	Not Reported
Hanekom et al 2012	71.6 ± 61.8	71.8 ±48.5	0.98	14.5 ± 11	17.1 ±± 14.1	0.2
Routsi et al 2010	9 (6–24)*	17 (6–30)*	0.23	Not reported	Not reported	Not reported
Brummel et al 2014	CT:5 (2.8–9.6)	4 (3–6.7)	0.67	CT: 7.9 (5.1–15)	8.6 (6–16.2)	0.46
	PT:3.5 (2.3–7.2)			PT: 7 (5–10.5)		

Values are presented in mean ± SD or median (P_25_-P_50_).

* median (min-max). CT. Cognitive therapy plus early physiotherapy. PT: Early physiotherapy

#### Time in rehabilitation after discharge

None of the included studies reported data on this outcome.

## Discussion

This systematic review and meta-analysis included seven studies (774 patients). Due to substantial heterogeneity of patients, interventions, and outcome timing/measures among studies of early rehabilitation in the ICU/HDU that were included, it was not possible to pool results for the primary outcome (i.e. functional status at ICU discharge) in this review. The included studies reported conflicting results on the effect of early rehabilitation on functional status at ICU discharge. However, early rehabilitation was associated with an increased probability of walking without assistance at hospital discharge. In addition, early rehabilitation was associated with improved distance walked at hospital discharge and a reduced risk of ICU-acquired weakness. Finally, no significant effects were found on the secondary outcomes of muscle strength, quality of life, duration of mechanical ventilation, and length of stay in the ICU or hospital.

All the included studies showed an improvement in functional status from baseline to next evaluation, but overall, there was no positive effect. Only Schweickert et al (2009) reported significant differences in the Barthel index compared to usual care, but patients in the usual care group of this study spent a longer time with limited mobility compared to other studies included in this analysis [35,36]. This could explain the significant difference in favour of the intervention group. These results differ with a previous systematic review [16] and a meta-analysis [17], which showed a significant difference in favour of early mobilisation on physical function. One possible reason for this discrepancy could be our strict definition of usual care.

According to our results, there was a potential improvement in distance walked at hospital discharge, but not at ICU discharge. This potential benefit was mainly driven by the results of Nava [30] and Burtin et al [31], where the components of early rehabilitation were more active (i.e. treadmill and cycloergometer in bed) and focused on the lower limbs. Patients in the study by Schweickert et al [32] walked fewer meters than individuals in other trials that assessed this outcome. This phenomenon could be explained by a shorter length of stay in ICU and in hospital, so patients had a shorter time of exposure to the intervention/usual care compared to subjects in other trials. In the study by Denehy et al [35], where was there no difference between groups in 6MWT at hospital discharge, patients in the control group walked greater distances at ICU discharge than those in the intervention group. However, the mean change in the distances walked between ICU and hospital discharge was higher in the intervention group. The lack of any detailed definition of usual care prevents further conclusions about possible factors that could have explained that difference.

Poor peripheral muscle strength has been associated with decreased walking distance in the 6-minute walking test [37], longer duration of mechanical ventilation [38], increased length of stay in ICU and higher mortality rates [39]. In this systematic review, no differences were found between groups when peripheral muscle strength was measured by handgrip strength. However, Burtin et al [31] found greater isometric quadriceps strength in the intervention group at hospital discharge which may account for the differences observed in the 6MWT in favour of experimental group. Schweickert et al [32] also found higher MRC scores in the intervention group but those were not significant. Although the MRC score has been shown to be reliable and valid [40], one difficulty in its application is the differentiation between a score of four (patient is able to move against gravity plus some external resistance) and five (patient is able to move limbs against gravity plus a maximal external resistance) [41], therefore, small differences can be missed due to the limitations of the instrument of measurement. The results for muscle strength are similar to those reported by Li et al [5]. In relation to ICUAW, there was evidence of a potential benefit of early rehabilitation, which is similar to the results reported by Hermans et al [12]. The variation in the dose and components of early rehabilitation (i.e. sedation interruption protocol, physical activity or electrical muscle stimulation), and timing for assessment limits the application of this finding.

Health related quality of life is a broad concept referring to how individuals rate their own existence and has been deemed as an important outcome for ICU survivors [24]. In this review, we were not able to find a significant difference between the intervention and control groups, which differs from the study by Kayambu et al [17] that reported improved quality of life in patients who received early mobilisation/rehabilitation during ICU stay. However, our findings are consistent with research on quality of life of ICU survivors [42,43], which has found that quality of life remains below the general population mean up to 12 months after hospital discharge. Severity of illness and poor quality of life before ICU admission have been deemed as predictors of physical functioning [42], but the lack of pre-morbid information about this outcome prevents generating conclusions about its influence on our results.

Duration of mechanical ventilation, length of stay in ICU and in hospital are health care utilisation outcomes. Previous systematic reviews have found positive effects of early mobilisation in the ICU using these measures [5,17,44], however we have not been able to support those results. From the two trials reporting information on duration of mechanical ventilation, only one showed significant shorter times for the intervention group [32]. However, results from both trials might have been highly influenced by an daily interruption protocol for sedation, which has been shown in other trials to be associated with shorter weaning times from mechanical ventilation and shorter length of stay in ICU [45]. Three trials reported a decreased length of stay in hospital in the intervention group [31,34,36], however these results should be interpreted carefully because other factors such as discharge policies and population case mix may explain the difference. Previous systematic reviews have found similar results [5,17,44].

It must be noted that the effect sizes reported are affected by the individual studies’ biases. Lack of appropriate concealment of randomisation schedule and blinding may lead to overestimation of results [29]. Blinding of patients and therapist is difficult to accomplish in physiotherapy clinical trials because the intervention is actively performed. While outcome assessor blinding is potentially possible, Burtin et al [31] and Nava [30] did not fulfil this criterion of methodological rigour. On the other hand, exclusion of patients from the analysis after randomisation and underpowered trials (recruitment was stopped early or they did not perform sample size calculation) might affect the possibility of finding an association [29], which could be the case for the trials by Nava [30], Burtin et al [31], Denehy et al [35] and Brummel et al [36].

Due to the strict inclusion and exclusion criteria defined for this systematic review, only one study examining the effect of electrical muscle stimulation during ICU stay was included, which had modest results. Nonetheless, two recent systematic reviews have been published, which suggest neuromuscular electrical stimulation could decrease muscle wasting in critically ill patients with lower acuity [46,47]. Studies assessing functional outcomes in a long term follow up are needed to support the use of this intervention in the ICU.

The results reported in this systematic review highlight the necessity of defining the most appropriate outcome measurements for critically ill patients in order to capture and monitor their health care needs through early rehabilitation. Hospital discharge and ambulatory follow-up visits at three, six and 12 months are recommended time points to measure the effect of the intervention in order to determine whether improvements are due to phenomena such as regression to the mean and natural recovery of the impairment, or due to early rehabilitation during ICU stay.

We only included studies in which usual care was defined, in line with European Society of Intensive Care task force recommendations [1]. However, all included studies defined usual care in a different way; ranging from standard medical care and physiotherapy ordered by primary care team [31,32,36] to frequent mobilisation including walking out of bed [34,35]. The components of the early rehabilitation were also dissimilar among the included studies. In every trial included in this systematic review, the early rehabilitation group performed activities out of bed, but the level of activity was different, ranging from transferring from bed to a chair [33] in the less active extreme to training on a treadmill [30] in the most active extreme. The variation in describing both usual care, and early rehabilitation limits our conclusion about the real differences among them and poses a challenge in the translation of these findings into practice [48].

One possible reason for variation of usual care used in different ICUs is the barriers and facilitators therapists find to carry out early rehabilitation. Leditschke et al [49] performed a qualitative audit during four weeks in a level III mixed ICU in Canberra, Australia, in order to determine reasons for not mobilising patients, and found that around 50% of reasons were avoidable. The most common ones were femoral position of vascular access, team coordination for procedures and sedation management. Bailey et al [50] highlighted that a cultural change is required to mobilise patients who are mechanically ventilated. Specifically, staff need to acknowledge the importance of patient-focused outcomes and teamwork, they need to improve collaboration and teamwork, create a reliable early mobility care pathway and recognise which current practices could interfere with mobility interventions. It should be noted that implementation is probably more dependent on particular issues within individual ICUs more than on universal issues, although teamwork is fundamental for the success of early rehabilitation.

Based on the data collected, a structured exercise program might be enough to improve outcomes in critically ill patients. The addition of daily interruption of sedation seems to increase the effect of early mobilisation. It is advisable to generate a consistent intervention program that includes safety criteria to start and interrupt sessions. When a cycloergometer or electrical muscle stimulation is available, they could be included as long as they do not put the patient at risk of an adverse event.

### Limitations

The studies included in this systematic review covered a wide variety of conditions and the descriptions of interventions were scant. In addition, definitions of usual care and early rehabilitation varied across the different studies, which implied more demanding sessions in some cases (e.g., Nava [30],Denehy et al. [35]). The question about the influence of frequency, duration, intensity, volume and timing of start upon functional status remains unanswered. More detailed information about control and active interventions are required to draw conclusions from statistical to clinical significance.

Type of outcomes, instruments used and timing for assessment were heterogeneous in the trials included, which limited the possibility of performing more meta-analyses.

It is not possible to draw conclusions about the influence of ICUAW over functional outcomes or the time in rehabilitation based on the pooled results, because none of the studies presented information for this subgroup of patients.

### Future research

Future studies should choose carefully the instruments and outcomes to be assessed. It would be advisable to measure peripheral muscle strength using the MRC score and physical capacity with the 6-minute walking distance test. Also a patient related outcome measure should be included, for instance health related quality of life, and health care utilisation outcomes (e.g. length of stay in ICU and hospital, days of mechanical ventilation, sessions of rehabilitation after discharge).

Considering the number and variety of ongoing studies, an update of this review in at least one year is advisable. In particular, those clinical trials testing electrical muscle stimulation (NCT0070909124 and ISRCTN35179428), the addition of cycloergometry to routine physiotherapy (DRKS00004347) and rehabilitation in populations with specific diagnosis such as sepsis (ACTRN 12610000808044) and COPD (NCT00628992) should provide useful results. Also, an individual patient data meta-analysis would yield valuable information in relation to characteristics of patients with ICUAW and their influence on functional status.

## Conclusions

Early rehabilitation during ICU stay has no effect on functional status, although it improves patients’ walking ability measured by proportion of individuals walking without assistance at hospital discharge. There is a tendency towards improved outcomes assessed by incidence of ICUAW and 6-minute walking test in those patients who received early mobilisation/ rehabilitation. Due to lack of information, it was not possible to accomplish our primary aim: to determine the impact of time from ICU admission to first mobilisation session and the dose of physical therapy on functional status.

Establishment of a standardised rehabilitation program during ICU stay would provide more benefit in those settings where currently there is no early intervention aiming to improve functional recovery. The available options for implementation are: physical therapy/occupational therapy and interruption of sedation, cycloergometer, physical and cognitive therapy, electrical muscle stimulation and programs of progressive exercise.

Consensus is necessary about which outcomes to assess, which instruments to use and which time points are relevant in order to improve the evidence in favour of early rehabilitation which would impact the production of evidence-based recommendations in future clinical practice guidelines.

Results from ongoing studies in this topic would give useful information to determine the real effect of different bundles of interventions.

## Supporting Information

S1 PRISMA Checklist PRISMA checklist. (DOCX)

S1 Supporting Information Search strategy. (DOCX)

S1 Table Characteristics of ongoing studies that would be eligible for this systematic review. (DOCX)

S2 Table Characteristics of excluded studies and reasons for exclusion. (DOCX)
