# Supplementary material for: Effect of Early Rehabilitation during Intensive Care Unit Stay on Functional Status: Systematic Review and Meta-Analysis
Source: PLoS One. 2015 Jul 1;10(7):e0130722. doi: 10.1371/journal.pone.0130722 (PMC4488896; doi:10.1371/journal.pone.0130722)
Supplement: S1 Supporting Information — (DOCX) [file pone.0130722.s002.docx]

## S1 Supporting information. Search strategy:

Search Strategy in OVID Medline In-Process & Other Non-Indexed Citations and Ovid MEDLINE(R) 1946 to Present:

1 Critical Care/

2 critical care.mp.

3 intensive care units/

4 intensive care unit?.mp.

5 burn units/

6 burn unit?.mp.

7 coronary care units/

8 coronary care unit?.mp.

9 recovery room/

10 recovery room?.mp.

11 respiratory care units/

12 respiratory care unit?.mp.

13 Critical Illness/rh [Rehabilitation]

14 Critical Illness/

15 (critical illness or critically ill).mp.

16 *Intensive Care/

17 intensive care.mp.

18 intensive treatment unit?.mp.

19 intensive therapy unit?.mp.

20 high dependency unit?.mp.

21 ICU.mp.

22 HDU.mp.

23 1 or 2 or 3 or 4 or 5 or 6 or 7 or 8 or 9 or 10 or 11 or 12 or 13 or 14 or 15 or 16 or 17 or 18 or 19 or 20 or 21 or 22

24 exp Rehabilitation/

25 rehabilitat*.mp.

26 exp Physical Therapy Modalities/

27 physical therapy modalit?.mp.

28 physical therap*.mp.

29 physiotherap*.mp.

30 kinesiotherap*.mp.

31 exp Exercise Therapy/

32 exercise therap*.mp.

33 physical exertion/

34 physical exertion.mp.

35 Early Ambulation/

36 Early Ambulation.mp.

37 mobilization.mp.

38 mobilisation.mp.

39 Muscle Weakness/rh [Rehabilitation]

40 Muscle Weakness/th

41 Neuromuscular Diseases/rh [Rehabilitation]

42 24 or 25 or 26 or 27 or 28 or 29 or 30 or 31 or 32 or 33 or 34 or 35 or 36 or 37 or 38 or 39 or 40 or 41

43 randomized controlled trial.pt.

44 controlled clinical trial.pt.

45 randomized.ab.

46 placebo.ab.

47 drug therapy.fs.

48 randomly.ab.

49 trial.ab.

50 groups.ab.

51 43 or 44 or 45 or 46 or 47 or 48 or 49 or 50

52 exp animals/ not humans.sh.

53 51 not 52

54 23 and 42 and 53
