# Supplementary material for: Effect of Early Rehabilitation during Intensive Care Unit Stay on Functional Status: Systematic Review and Meta-Analysis
Source: PLoS One. 2015 Jul 1;10(7):e0130722. doi: 10.1371/journal.pone.0130722 (PMC4488896; doi:10.1371/journal.pone.0130722)
Supplement: S1 Table — (DOCX) [file pone.0130722.s003.docx]

*S1 Table. Characteristics of ongoing studies that would be eligible for this systematic review*

| Name of trial/ Identifier | Population | Intervention | Control | Outcomes* | Estimated date of completion |
| --- | --- | --- | --- | --- | --- |
| Early rehabilitation in critical care (eRiCC): functional electrical stimulation with cycling protocol for a randomised controlled trial. ACTRN 12612000528853. Protocol: BMJ Open. 2012;2(5) | Patients with | Usual PT + | Usual PT including | PFIT |  |
|  | sepsis, mechanical | cycoergometer for 1h, | respiratory care and | FSS-ICU |  |
|  | ventilation > 48 | 5 times a week + FES | mobilisation. Up to 15 | 6MWDT | Not |
|  | hrs, expected to | over gluteal, | min each session | MRC score | reported |
|  | be ≥ 4 days in ICU | hamstring, quadriceps and gastrocnemius |  | Handheld dynamometry | Estimated date first |
|  |  | muscles of one lower |  | LOS in ICU | enrolment: |
|  |  | limb |  | LOS in hospital | 30/05/2012 |
|  |  |  |  | Days in mechanical ventilation |  |
| Early rehabilitation in sepsis: a prospective randomised controlled trial investigating functional and physiological outcomes The i-PERFORM Trial. ACTRN 12610000808044. Protocol: BMC Anesthesiology. 2011; 11:21 | Patients with sepsis, mechanical | 30 min, 1 or 2 times a day. EMS, passive and | Usual care (i.e. sitting out of bed, walking) | Acute care index of function |  |
|  | ventilation > 48 | active mobilisation, |  | PFIT | Not |
|  | hrs | siting out of bed, arm |  | SF-36 | reported |
|  |  | and leg ergometry, |  | LOS in ICU | Estimated |
|  |  | ambulation |  | LOS in hospital | date first |
|  |  |  |  | Days in mechanical ventilation | enrolment: 01/09/2010 |
| Early Rehabilitation of COPD Patients in ICU. NCT00628992 | Patients with COPD, intubated | Group_1_: EMS in thigh, 5 times a week | Standard care | Manual muscle testing | March 2013 |
|  | receiving | Group_2_: |  | LOS in ICU |  |
|  | mechanical ventilation, | Cycloergometer, 5 times a week |  | LOS in hospital |  |
|  | conscious and cooperative. | Group_3_: Cycloergometer + EMS in thigh, 5 times a week |  |  |  |
| Effect of post-operative early mobilisation on pulmonary complications and hospital stay: a randomised trial. ISRCTN28048472 | Elective | Daily progressive | Routine PT | LOS in ICU | October |
|  | abdominal surgery requiring HDU | mobility program (i.e. sitting out of bed, progressive ambulation) |  | LOS in hospital | 2013 |
| Effects of an early, combined endurance and resistance training on mechanically ventilated, critically ill patients – a randomised controlled trial. DRKS00004347 | Patients expected | Passive/active training | Routine PT (i.e. | FIM |  |
|  | to be > 72 hours | with cycloergometer. | passive and active | Time up and go | Not |
|  | in mechanical | Resistance training, | mobilisation and | 6MWDT | reported |
|  | ventilation | mobilisation and | activities of daily | MRC score | Estimated |
|  |  | activities of daily living | living) | Handgrip dynamometry | date first enrolment: |
|  |  |  |  | Quadriceps strength | 10/10/2010 |
|  |  |  |  | SF-36 |  |
| The effects of electrical muscle stimulation on muscle mass, strength and function in patients receiving mechanical ventilation: a randomised controlled, single blind feasibility study. ISRCTN35179428 | Patients expected | Usual rehabilitation + | Usual rehabilitation + | Barthel Index | January |
|  | to be > 72 hours | EMS in biceps brachii | Sham EMS in biceps | 6MWDT | 2014 |
|  | in mechanical ventilation | and quadriceps femoris. Twice daily, | brachii and quadriceps femoris. | Manual muscle testing |  |
|  |  | 30 min each session | Twice daily, 30 min each session | Handgrip dynamometry |  |
|  |  |  |  | Handheld dynamometry |  |
|  |  |  |  | SF-36 v2 |  |
|  |  |  |  | LOS in ICU |  |
|  |  |  |  | LOS in hospital |  |
| A randomised controlled trial of intensive versus standard physical rehabilitation therapy in the critically ill : Extra Physiotherapy In Critical Care. ISRCTN20436833 | Patients admitted to surgical or medical ICU, receiving invasive or non-invasive mechanical ventilation > 48 hrs | 5 times a week routine PT + individualised structured exercises program | 5 times a week routine PT, including retraining session | Quality of life | July 2014 |
| Standardized Rehabilitation for Intensive Care Unit (ICU) Patients With Acute Respiratory Failure. NCT00976833 | Patients with lung injury in | Daily passive mobilisation, physical | Usual Care | Functional capacity | October 2014 |
|  | mechanical | therapy and |  | Quality of life |  |
|  | ventilation (invasive or non-invasive) | resistance training |  | LOS in hospital |  |
| The Impact of Early Mobilization Protocol in Patients in the ICU. NCT01769846 | Patients in | Usual mobilisation + | Usual mobilisation by | FSS-ICU | December |
|  | mechanical | Cycloergometer. In a | ICU team | MRC score | 2015 |
|  | ventilation < 48 | passive way at 20 |  | Quality of life |  |
|  | hrs, expected 24 | cycles/min for |  | LOS in ICU |  |
|  | hrs more. Stable | sedated patients. |  | Days in |  |
|  | cardiorespiratory condition | Cooperative patients: 20 min of active cycling at individually adjusted intensity. 5 times a week |  | mechanical ventilation |  |
| Neuromuscular electrical stimulation for intensive care unit-acquired weakness: protocol and methodological implications for a randomized, sham-controlled, phase II trial. NCT00709124. Protocol: Phys Ther. 2012 Dec; 92(12): 1564-79. | Patients in | Daily routine PT + 60 | Daily routine PT + | FSS-ICU | July 2013 |
|  | mechanical ventilation for 1 | min NMES over quadriceps, tibialis | sham NMES | Manual muscle testing |  |
|  | day, expected 2 more days | anterior and gastrocnemius |  | Handheld dynamometry |  |
|  |  |  |  | Handgrip dynamometry |  |
|  |  |  |  | LOS in ICU |  |
|  |  |  |  | LOS in hospital |  |
|  |  |  |  | Days in mechanical ventilation |  |
| Dose of Early Therapeutic Mobility: Does Type or Frequency Matter? (ETM). NCT00999011 | Mechanical ventilation ≥ 24 | Low intensity: in-bed activities, passive and | Low intensity: in-bed activities, passive and | Handgrip dynamometry | May 2012 |
|  | hrs, expected to | active range of | active range of | MRC score |  |
|  | continue for 24 | motion with backrest | motion with backrest | LOS in UCI |  |
|  | hrs more. Stable cardiorespiratory | elevation > 40 degrees | elevation > 40 degrees. | Days in mechanical |  |
|  | state | Moderate intensity: sitting at the edge of bed, passive transfer to chair, standing transfer to chair and standing or walking. | Moderate intensity: sitting at the edge of bed, passive transfer to chair, standing transfer to chair and standing or walking. | ventilation |  |
|  |  | Twice daily for 20 min each session. Intensity according to ability to follow commands and to move limbs against gravity | Once daily for 20 min each session. Intensity according to ability to follow commands and to move limbs against gravity |  |  |
