# Supplementary material for: Effect of Early Rehabilitation during Intensive Care Unit Stay on Functional Status: Systematic Review and Meta-Analysis
Source: PLoS One. 2015 Jul 1;10(7):e0130722. doi: 10.1371/journal.pone.0130722 (PMC4488896; doi:10.1371/journal.pone.0130722)
Supplement: S2 Table — (DOCX) [file pone.0130722.s004.docx]

*S2 Table. Characteristics of excluded studies and reasons for exclusion.*

| Author | Title | Reference | Reason for exclusion |
| --- | --- | --- | --- |
| Paratz JD, Stockton K, Plaza A, Muller M, Boots RJ. | Intensive exercise after thermal injury improves physical, functional, and psychological outcomes | Journal of Trauma & Acute Care Surgery. 2012;73(1):186-94 | Usual Care did not fulfil definition |
| Karatzanos E, Gerovasili V, Zervakis D, Tripodaki ES, Apostolou K, Vasileiadis I, et al. | Electrical muscle stimulation: an effective form of exercise and early mobilization to preserve muscle strength in critically ill patients | Critical Care Research & Practice. 2012;2012:432752 | Duplicated report |
| Paternostro-Sluga T, Gruther W. | Intensive physical therapy reduces length of hospital stay in critically ill patients. | PM and R. 2012 October; 1: S310-S311. | Conference abstract |
| Yohannan SK, Tufaro PA, Hunter H, Orleman L, Palmatier S, Sang C, et al | The utilization of Nintendo Wii™ during burn rehabilitation: a pilot study. | Journal of Burn Care & Research. 2012 Jan-Feb; 33(1):36-45. | No data available |
| Caruso FCR, Arena R, Mendes RG, Reis MS, Papa V, Borghi-Silva A. | Heart rate autonomic responses during deep breathing and walking in hospitalised patients with chronic heart failure | Disability and Rehabilitation. 2011;33(9):751-7 | No relevant outcomes reported |
| Chen SY, Su CL, Wu YT, Wang LY, Wu CP, Wu HD, et al. | Physical training is beneficial to functional status and survival in patients with prolonged mechanical ventilation. | Journal of the Formosan Medical Association. 2011 Sep; 110(9):572-9. | Usual Care did not fulfil definition |
| Denehy L, Berney S, Skinner E, Edbrooke L, Haines K, Warrillow S, et al. | Evaluation of exercise rehabilitation for survivors of intensive care: An assessor blinded randomised controlled trial | American Journal of Respiratory and Critical Care Medicine. 2011; 183 (Meeting Abstracts):[A2642] | Duplicated report |
| Evans J, Tsekouras C, Johnson K, Marx M | Effect of Early Mobilization Efforts on Postoperative Length of Stay After Cardiac Transplant and Left Ventricular Assist Device Surgery | Critical Care Nurse. 2011;31(2):e50-1 | Not a clinical trial |
| Karatzanos L, Gerovasili V, Zervakis D, Dimopoulos S, Tripodaki EA, Pitsolis T. | Electrical Muscle Stimulation Is An Effective Form Of Exercise And Early Mobilization In ICU Patients | American Journal of Respiratory and Critical Care Medicine. 2011; 183(Meeting Abstracts):[A3740] | Duplicated report |
| Appleton R. | Early physical and occupational therapy in mechanically ventilated medical patients improves return to independent functional status at hospital discharge. | Journal of the Intensive Care Society. 2010 July;11(3):202-3 | Editorial or comment on an article |
| Brahmbhatt N, Murugan R, Milbrandt EB. | Early mobilization improves functional outcomes in critically ill patients. | Critical Care. 2010;14(5) | Editorial or comment on an article |
| Charet GP | Patient care. To reduce ICU stays, get patients moving | Hospitals & health networks / AHA. 2010 Aug; 84(8):14 | Editorial or comment on an article |
| Hanekom S, Louw Q, Coetzee A. | Physiotherapy management of critically ill patients guided by an evidence based protocol is safe and effective: A preliminary study. | Intensive Care Medicine. 2010 September; 36: S324. | Duplicated report |
| Mendes RG, Simoes RP, De Souza Melo Costa F, Pantoni CB, Di Thommazo L, Luzzi S, et al. | Short-term supervised inpatient physiotherapy exercise protocol improves cardiac autonomic function after coronary artery bypass graft surgery—a randomised controlled trial. | Disability and rehabilitation. 2010; 32(16):1320-7. | Usual Care did not fulfil definition |
| Needham DM, Korupolu R, Zanni JM, Pradhan P, Colantuoni E, Palmer JB, et al. | Early physical medicine and rehabilitation for patients with acute respiratory failure: a quality improvement project | Archives of Physical Medicine and Rehabilitation. 2010 Apr; 91(4):536-42. | Not a clinical trial |
| Patel B, Poston J, Pohlman A, Hall JB, Kress JP. | Complications of critical illness in mechanically ventilated patients in a randomized controlled trial of early mobilization. | American Journal of Respiratory and Critical Care Medicine. 2010 01 May; 181 (1 Meeting Abstracts). | Editorial or comment on an article |
| Troosters T, Probst VS, Crul T, Pitta F, Gayan-Ramirez G, Decramer M, et al. | Resistance training prevents deterioration in quadriceps muscle function during acute exacerbations of chronic obstructive pulmonary disease. | American journal of respiratory and critical care medicine. 2010; 181(10):[1072] | Clinical setting was not an ICU/HDU |
| Forgiarini Junior LA, de Carvalho AT, Ferreira TS, Monteiro MB, Bosco AD, Goncalves MP, et al. | Physical therapy in the immediate postoperative period after abdominal surgery. | Jornal Brasileiro de Pneumologia. 2009 May; 35(5):455-9. | No relevant outcomes reported |
| Jakob SM, Takala J. | Physical and occupational therapy during sedation stops. | Lancet. 2009 May 30; 373(9678):1824-6. | Editorial or comment on an article |
| Jarden M, Baadsgaard M, Hovgaard D, Boesen E, Adamsen L. | A randomized trial on the effect of a multimodal intervention on physical capacity, functional performance and quality of life in adult patients undergoing allogeneic SCT. | Bone Marrow Transplantation. 2009; 43(9):725-37. | Clinical setting was not an ICU/HDU |
| Needham DM, Chandolu S, Zanni J. | Interruption of sedation for early rehabilitation improves outcomes in ventilated, critically ill adults. | Australian Journal of Physiotherapy. 2009;55(3):210 | Editorial or comment on an article |
| Schweickert WD, Poston JT, Esbrook CL, Pawlik AJ, Pohlman AS, Hall JB. | Temporal relation of early mobilization on recovery of functional independence in mechanically ventilated patients | American Thoracic Society International Conference, May 15-20, 2009, San Diego. 2009: [A2168]. | Duplicated report |
| Morris PE, Goad A, Thompson C, Taylor K, Harry B, Passmore L, et al | Early intensive care unit mobility therapy in the treatment of acute respiratory failure. | Critical Care Medicine. 2008 Aug; 36(8):2238-43. | Not a clinical trial |
| De Morton NA, Keating JL, Berlowitz DJ, Jackson B, Lim WK. | Additional exercise does not change hospital or patient outcomes in older medical patients: a controlled clinical trial. | Australian Journal of Physiotherapy. 2007; 53(2):105-11. | Clinical setting was not an ICU/HDU |
| Chiang LL, Wang LY, Wu CP, Wu HD, Wu YT. | Effects of physical training on functional status in patients with prolonged mechanical ventilation. | Physical therapy. 2006 Sep; 86(9):1271-81. | Usual Care did not fulfil definition |
| Porta R, Vitacca M, Gile LS, Clini E, Bianchi L, Zanotti E, et al. | Supported arm training in patients recently weaned from mechanical ventilation. | Chest. 2005 Oct; 128(4):2511-20. | No relevant outcomes reported |
| Delaney CP, Zutshi M, Senagore AJ, Remzi FH, Hammel J, Fazio VW. | Prospective, randomized, controlled trial between a pathway of controlled rehabilitation with early ambulation and diet and traditional postoperative care after laparotomy and intestinal resection. | Diseases of the colon and rectum. 2003 Jul; 46(7):851-9. | Rehabilitation did not fulfil definition |
| Zanotti E, Felicetti G, Maini M, Fracchia C. | Peripheral muscle strength training in bed-bound patients with COPD receiving mechanical ventilation: effect of electrical stimulation. | Chest. 2003 Jul; 124(1):292-6. | No relevant outcomes reported |
| Hayes MJ, Morris GK, Hampton JR. | Comparison of mobilization after two and nine days in uncomplicated myocardial infarction. | British Medical Journal. 1974 Jul 6; 3(5922):10-3. | Clinical setting was not an ICU/HDU |
| Brummel et al | ACT-ICU Study: Activity and Cognitive Therapy in the Intensive Care Unit | NCT01270269 | Duplicated report |
| Kress et al | Early Directed Physical Therapy in the Management of Mechanically Ventilated Patients in a Medical Intensive Care Unit | NCT00322010 | Duplicated report |
| Gosselink et al | Early Exercise Training in Critically Ill Patients | NCT00695383 | Duplicated report |
| Skafte et al | Early Mobilization in Intensive Therapy | NCT01549808 | Not a clinical trial  No relevant outcomes reported |
| Nanas et al | Electrical Muscle Stimulation (EMS), a Preventive and Therapeutic Tool for Critical Illness Polyneuromyopathy (CIPNM) | NCT00882830 | Duplicated report |
| Parry et al | eRiCC trial: Early rehabilitation in Critical Care | ACTRN12612000528853 | Duplicated report |
| Paratz et al | Exercise in Critically Ill Patients With Sepsis | NCT01364909 | No relevant outcomes reported |
| Kayambu et al | Mobilising Critically Ill patients: Physiological and Functional Outcomes following Early Rehabilitation in Sepsis | ACTRN12610000808044 | Duplicated report |
| Hart et al | Rehabilitation Following Critical Illness | NCT00976807 | Clinical setting was not an ICU/HDU |
| Tidswell et al | Safety and Performance of Muscle Activation for Critical Care Patients | NCT01552616 | Rehabilitation did not fulfil definition |
| Eikermann et al | Systematic Team Approach to Guide Early Mobilization in Surgical Intensive Care Unit Patients | NCT01363102 | No relevant outcomes reported |
| Denehy et al | Evaluation of exercise rehabilitation for survivors of intensive care | ACTRN12605000776606 | Duplicated report |
| Poulsen et al | Transcutaneous Electric Muscle Stimulation (TEMS) in Septic Patients | NCT01071343 | Subject was his/her own control |
| Moss et al | Treatment of Critical Illness Polyneuromyopathy (Do it now) | NCT01058421 | Not clear if clinical setting was ICU/HDU |
| Kho et al | Use of Neuromuscular Electrostimulation (NMES) for Treatment or Prevention of ICU-Associated Weakness | NCT00709124 | Duplicated report |
| Maturana et al | Use of two physiotherapeutic techniques in the patient admitted to the intensive care unit | RBR-6sz5dj | Rehabilitation did not fulfil definition |
| Kress et al | Early Mobilization in the ICU | NCT01777035 | No relevant outcomes reported |
| Laterre et al | Effect of Early Physical Activity on Skeletal Muscle Signalling Pathways Controlling Protein Turnover in Patients With Sepsis | NCT01787045 | No relevant outcomes reported |
